# CPAP Use and Retinal Disease Risk in Obstructive Apnea: A Cohort Study

**DOI:** 10.3390/vision9030065

**Published:** 2025-08-01

**Authors:** Dillan Cunha Amaral, Pedro Lucas Machado Magalhães, Muhammad Alfatih, Bruna Gabriel Miranda, Hashem Abu Serhan, Raíza Jacometti, Bruno Fortaleza de Aquino Ferreira, Letícia Sant’Ana, Diogo Haddad Santos, Mário Luiz Ribeiro Monteiro, Ricardo Noguera Louzada

**Affiliations:** 1 Department of Ophthalmology and Otorhinolaryngology, Faculty of Medicine, Federal University of Rio de Janeiro, Rio de Janeiro 21941-853, RJ, Brazil; 2 Faculty of Medicine, Institute of Medical Education, Angra dos Reis 23914-360, RJ, Brazil; pedrolucasm@mail.tau.ac.il; 3 Faculty of Medicine, Universitas Indonesia, Jakarta 16424, Indonesia; muhammad.alfatih01@ui.ac.id; 4 Faculty of Medicine, Federal University of Catalão, Catalão 75705-220, GO, Brazil; bruna.miranda@ufcat.edu.br; 5 Department of Ophthalmology, Hamad Medical Corporation, Doha P.O. Box 3050, Qatar; habuserhan@hamad.qa; 6 Department of Ophthalmology and Otorhinolaryngology, Faculty of Medicine, University of São Paulo, São Paulo 05508-220, SP, Brazil; raizajacometti@usp.br (R.J.); brunofortaleza@alumni.usp.br (B.F.d.A.F.); mario.monteiro@fm.usp.br (M.L.R.M.); 7 Hospital Alemão Oswaldo Cruz, São Paulo 01323-020, SP, Brazil; leticiasantc@gmail.com (L.S.); dhaddadsantos@gmail.com (D.H.S.); 8 Laboratory for Investigation in Ophthalmology (LIM-33), Division of Ophthalmology, Faculty of Medicine, University of São Paulo, São Paulo 01246-903, SP, Brazil

**Keywords:** obstructive sleep apnea, CPAP therapy, retinal diseases, diabetic retinopathy

## Abstract

Obstructive sleep apnea (OSA) is a common condition associated with intermittent hypoxia, systemic inflammation, and vascular dysfunction; mechanisms implicated in retinal disease pathogenesis. This real-world retrospective cohort study used data from the TriNetX Research Network to assess whether continuous positive airway pressure (CPAP) therapy reduces retinal disease incidence among adults with OSA and BMI between 25.0 and 30.0 kg/m^2^. After 1:1 propensity score matching, 101,754 patients were included in the analysis. Retinal outcomes included diabetic retinopathy (DR), age-related macular degeneration (AMD), retinal vein occlusion (RVO), and central serous chorioretinopathy (CSC). CPAP use was associated with a modest but statistically significant reduction in DR (3.2% vs. 3.4%, RR: 0.922, *p* = 0.016) and AMD (2.1% vs. 2.3%, RR: 0.906, *p* = 0.018), while no significant differences were found for RVO or CSC. These findings support prior evidence linking CPAP to improved retinal microvascular health and suggest a protective effect against specific retinal complications. Limitations include a lack of data on CPAP adherence, OSA severity, and imaging confirmation. Still, this study highlights the importance of interdisciplinary care between sleep and eye health, and the need for further prospective studies to validate CPAP’s role in preventing retinal disease progression in OSA patients.

## 1. Introduction

Obstructive sleep apnea (OSA) is a highly prevalent disorder characterized by recurrent episodes of upper airway collapse during sleep, leading to frequent arousals and often resulting in blood oxygen desaturation that drives sympathetic activation, oxidative stress, and systemic endothelial dysfunction. Studies indicate that the prevalence of OSA varies widely depending on the population studied and the diagnostic criteria used, with high rates observed in certain groups, such as aging populations and those with cardiometabolic comorbidities, precisely the profile of many patients seen in ophthalmic practice [1,2]. The retina and choroid are uniquely vulnerable targets of hypoxia-reperfusion injury, inflammatory signaling, autonomic dysregulation, and microvascular tone instability, which are all biologic sequelae linked to OSA. These pathways plausibly converge on several major retinal diseases. In diabetes, intermittent hypoxia and endothelial stress may amplify capillary dropout, leakage, and VEGF signaling, accelerating diabetic retinopathy (DR) progression. Recent large-scale electronic health record (EHR) data indicate that OSA is associated with higher rates of vision-threatening DR events and ocular interventions [2,3].

Choroidal circulatory compromise and inflammatory modulation have been implicated in age-related macular degeneration (AMD), and emerging EHR-based cohort work suggests individuals with diagnosed OSA experience increased AMD incidence, progression, and treatment needs over multi-year follow-ups. Experimental and imaging studies further indicate that continuous positive airway pressure (CPAP) therapy can alter posterior segment perfusion, including measurable changes in choroidal thickness, supporting a biologic link between nocturnal respiratory stability and macular health [2,4,5].

Other retinal vascular and choroidally mediated disorders have also been explored. Case–control and database studies report higher rates of retinal vein occlusion (RVO) and central serous chorioretinopathy (CSC) in patients with OSA, although data are mixed and frequently limited by sample size, confounding, or lack of treatment information [6,7].

Given the potential impact of OSA on ocular health, particularly regarding the development of retinal diseases, it is important to investigate whether appropriate treatment of OSA through continuous use of CPAP may exert a protective effect against the onset or progression of these pathologies. Recent studies suggest that regular CPAP use can improve vascular parameters and reduce systemic inflammation, factors that could decrease the risk of structural and functional retinal alterations [8]. Furthermore, evidence suggests that vascular dysfunction and alterations in the retinal nerve fiber layer, commonly observed in patients with OSA, may be mitigated by CPAP therapy, underscoring the importance of early and continuous intervention [1,9].

Despite increasing recognition of the association between OSA and retinal disease, studies assessing the role of CPAP treatment in modifying this risk remain limited. Leveraging the global TriNetX federated EHR research network, we evaluated whether documented CPAP use in adults with OSA was associated with differences in recorded diagnoses of DR, AMD, RVO, and CSC.

We hypothesized that patients with documented CPAP exposure would show lower recorded rates of microvascular and choroidally mediated retinal disease relative to matched OSA patients without CPAP documentation.

## 2. Materials and Methods

### 2.1. Data Source

This study adheres to the Strengthening the Reporting of Observational Studies in Epidemiology (STROBE) guidelines for cohort studies [10]. Institutional review board (IRB) approval and informed consent were not required, as the analysis was conducted using de-identified, aggregated data from a publicly available federated research database. No direct interaction or intervention with human participants occurred, and all de-identification measures were implemented in accordance with the Health Insurance Portability and Accountability Act (HIPAA) Privacy Rule. Data were collected from the TriNetX Analytics Network, a global federated health research platform that provides access to anonymized EHR from approximately 200 million patients across 160 healthcare organizations in 21 countries [11]. TriNetX supports large-scale retrospective analyses using structured clinical data, including diagnoses, procedures, laboratory values, medications, and demographics.

### 2.2. Study Design

This retrospective cohort study was conducted using data extracted on 22 February 2025. Adult patients aged 18 years or older with a diagnosis of OSA and a body mass index (BMI) between 25 and 30 kg/m^2^ were included. We limited inclusion to patients with this range BMI to reduce confounding from severe obesity, which clusters with advanced metabolic disease and variable access patterns that are incompletely captured in aggregated EHR data. OSA prevalence increases with excess body weight, but pathophysiology and care pathways in class II/III obesity may differ materially; thus, our findings are most applicable to an overweight OSA population commonly encountered in general medical and ophthalmic settings [2,3]. Patients were stratified into two cohorts based on documentation of CPAP therapy. The CPAP cohort included patients with a history of CPAP use, identified by procedure codes for CPAP initiation and management (UMLS:CPT:94660 or UMLS:SNOMED:47545007), along with a diagnosis of OSA (UMLS:ICD10CM:G47.33). The platform does not expose device-verified usage duration, nightly adherence, pressure settings, or home durable medical equipment fills [2,4]. The non-CPAP cohort included patients with OSA and BMI in the same range but with no record of CPAP use. The index date was defined as the first date on which a patient met all the criteria for their respective cohort. Patients whose index event occurred more than 20 years before the analysis date were excluded. Outcomes were analyzed within a time window that began 90 days after the index date and ended 7300 days (~20 years) after the index date or the last recorded encounter, whichever came first.

All diagnoses and procedures were required to be confirmed in structured EHR entries, and data quality is maintained across TriNetX partners through standardized validation protocols aligned with HIPAA. All reported counts, risk metrics, and *p*-values are those generated at the time of the original query (22 February 2025).

### 2.3. Outcome Definition

The primary outcomes were the incidence of retinal diseases, including DR, CSC, AMD, and RVO. DR was defined by the following ICD-10-CM codes: E11.311, E11.319, E11.329, and E11.359. CSC was defined by H35.71, H35.719, H35.712, H35.711, and H35.713. AMD was defined by H35.30, H35.31, and H35.32. RVO was defined by H34.81, H34.819, and H34.839. For all outcomes, patients with a record of the diagnosis prior to the index date were not excluded, and risk analysis included patients regardless of prior disease status.

### 2.4. Statistical Analysis

Propensity score matching (PSM) was performed in a 1:1 ratio within the TriNetX Advanced Analytics environment to balance the baseline characteristics of the two cohorts. Variables used for matching included age at index, sex, presence of diabetes mellitus, hypertensive diseases, chronic kidney disease (CKD), and BMI (continuous). Cohort characteristics before and after matching were evaluated using standardized mean differences, with values below 0.1 considered acceptable. Following PSM, both cohorts included 50,877 patients. Risk analysis was conducted to calculate the proportion of patients with each outcome, as well as the risk difference, risk ratio, and odds ratio, along with 95% confidence intervals. *p*-values were calculated using z-tests. Kaplan–Meier survival analysis was also performed using daily time intervals, with patients censored at their last recorded clinical event. All statistical analyses were conducted within the TriNetX platform, and the analyses were based on the data available as of 22 February 2025.

## 3. Results

**Figure 1:**
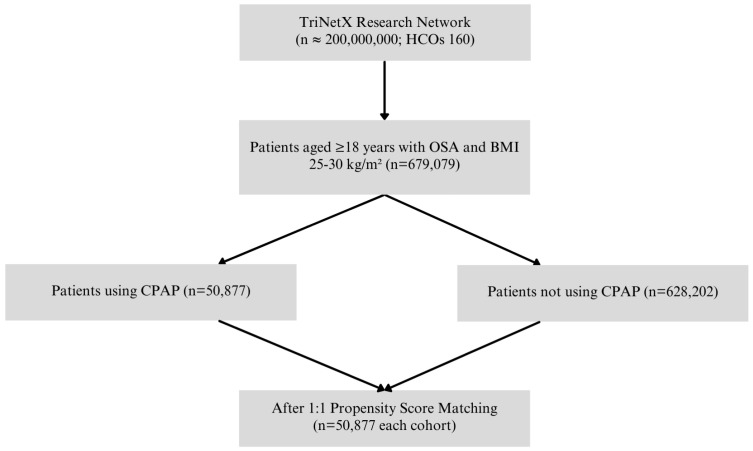
Patient selection flowchart.

A total of 679,079 adult patients with OSA and a body mass index (BMI) between 25.00 and 30.00 kg/m^2^ were identified from the TriNetX Research Network. Of these, 50,877 patients had documented CPAP use and were assigned to the CPAP-treated cohort, while 628,202 patients without CPAP documentation comprised the non-CPAP cohort. Following 1:1 propensity score matching, 50,877 patients remained in each group and were included in the final analysis. The patient selection process is illustrated in Figure 1.

**Figure 2:**
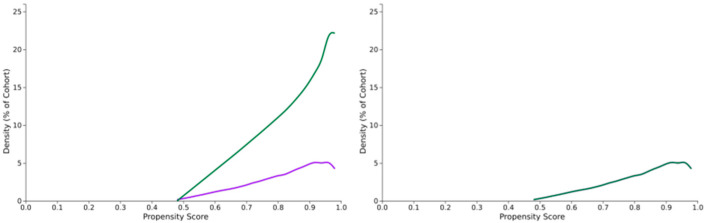
Propensity score distribution before and after matching. Left Panel: Propensity Score Distribution Before Matching. The treated (purple) and control (green) groups show considerable imbalance across propensity scores, indicating a mismatch in covariate distributions. Right Panel: Propensity Score Distribution After Matching. The groups have been balanced through matching, with overlapping distributions suggesting improved comparability between treated and control groups.

Baseline demographic and clinical characteristics were well balanced between the two matched cohorts (Table 1). The mean age at index date was 66.8 ± 13.8 years in the CPAP group and 66.9 ± 13.6 years in the non-CPAP group. Both groups had 66.0% male participants. The prevalence of diabetes mellitus was 47.9% in the CPAP cohort and 47.6% in the non-CPAP cohort. Hypertension was reported in 82.4% and 82.6% of patients in the CPAP and non-CPAP cohorts, respectively. CKD was present in 35.7% and 35.4% of patients, respectively. Mean BMI was similar across both groups (29.3 ± 3.6 kg/m^2^ vs. 29.3 ± 3.2 kg/m^2^). The mean follow-up duration was 966.9 ± 1031.4 days in the CPAP cohort and 1106.5 ± 1111.0 days in the non-CPAP cohort, with corresponding medians of 597 days (IQR: 1373) and 744 days (IQR: 1434). Propensity score distributions before and after matching are shown in Figure 2.

The incidence of retinal outcomes is summarized in Table 2. For the primary outcome of DR, the incidence was 3.2% in the CPAP group (*n* = 1611) compared to 3.4% in the non-CPAP group (*n* = 1748), yielding a risk difference of −0.3% (95% CI: −0.5 to −0.0; *p* = 0.016), with a risk ratio of 0.922 (95% CI: 0.862–0.985) and an odds ratio of 0.919 (95% CI: 0.858–0.985), favoring the CPAP group.

In the case of CSC, the incidence was extremely low and similar between groups (0.1% in both; 33 cases in the CPAP group vs. 32 cases in the non-CPAP group). There was no statistically significant difference (risk difference: 0.0%; RR: 1.031; 95% CI: 0.634–1.677; *p* = 0.901).

For AMD, incidence was 2.1% in the CPAP cohort (*n* = 1057) and 2.3% in the non-CPAP cohort (*n* = 1167), with a risk difference of −0.2% (95% CI: −0.4 to −0.0; *p* = 0.018), a risk ratio of 0.906 (95% CI: 0.834–0.983), and an odds ratio of 0.904 (95% CI: 0.831–0.983). RVO occurred in 141 patients (0.3%) in the CPAP group and 158 patients (0.3%) in the non-CPAP group. No statistically significant difference was observed (risk difference: −0.0%; RR: 0.892; 95% CI: 0.711–1.120; *p* = 0.325).

**Figure 3:**
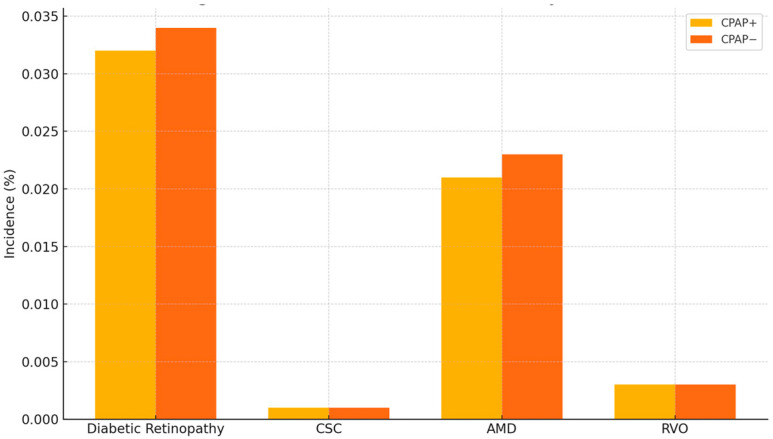
Incidence of retinal outcomes in CPAP+ vs. CPAP− cohorts.

**Figure 4:**
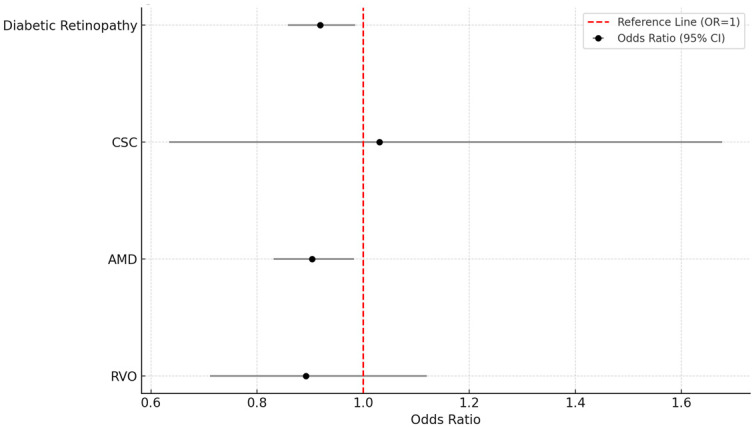
Odds ratios and 95% confidence intervals for retinal outcomes.

All results were derived from analysis of matched cohorts using risk analysis as provided by the TriNetX platform. Figure 3 presents a bar chart comparing the incidence of DR and AMD between cohorts, demonstrating a modest but consistent reduction in both outcomes among CPAP users. Figure 4 displays a comparative bar chart of odds ratios with 95% confidence intervals for all retinal outcomes, further supporting a lower risk of DR and AMD in the CPAP-treated group compared to those without CPAP.

## 4. Discussion

This large real-world retrospective cohort study used data from the TriNetX Research Network to investigate the impact of CPAP therapy on retinal outcomes in adults with OSA and elevated BMI. After 1:1 propensity score matching, the final analysis included 101,754 patients equally divided between CPAP users and non-users. CPAP use was associated with a modest but statistically significant reduction in the incidence of DR and AMD, while no significant associations were found for central serous chorioretinopathy or retinal vein occlusion.

### 4.1. Pathways by Disease

The heterogeneous pattern of associations we observed, i.e. modest risk reductions for DR and AMD but null findings for RVO and CSC, aligns with mechanistic expectations. Intermittent hypoxia, oxidative stress, and endothelial injury in OSA can amplify diabetic microvascular leakage and inflammatory cascades that accelerate DR progression; large EHR-based analyses have linked OSA to higher rates of vision-threatening DR events, underscoring biologic plausibility [2,3]. Choroidal perfusion instability and inflammatory signaling have been implicated in AMD pathogenesis, and individuals with diagnosed OSA show increased AMD incidence and progression in multi-system data. CPAP has been shown to modify retinal vascular parameters and to increase choroidal thickness in treated OSA, suggesting that nocturnal airway stabilization may partly restore posterior segment perfusion [2,3,4,12].

These mechanisms contribute to microvascular impairment and are implicated in the pathogenesis of DR and AMD [13]. Gavrilin et al. demonstrated that OSA disrupts nitric oxide bioavailability and enhances pro-inflammatory cytokine expression, leading to endothelial dysfunction and impaired autoregulation of vascular beds, including the retina [13]. In a randomized controlled trial, Turnbull et al. reported that CPAP therapy improved retinal microvascular function, emphasizing the potential of this intervention to counteract OSA-induced vascular damage [14]. These observations are further supported by Cristescu et al., who described a spectrum of ocular pathologies in OSA patients, highlighting the clinical relevance of retinal vascular compromise in this population [15].

In our matched cohort analysis, CPAP use was associated with a lower incidence of DR (3.2% vs. 3.4%; RR: 0.922; *p* = 0.016) and AMD (2.1% vs. 2.3%; RR: 0.906; *p* = 0.018). These findings are consistent with evidence from imaging-based studies. Tejero-Garcés et al. used optical coherence tomography to show microstructural improvements in the retina of OSA patients following CPAP initiation, while Lin et al. demonstrated enhanced retinal thickness and visual sensitivity post-therapy [8,16]. Moreover, a multicenter randomized controlled trial by West et al. found that CPAP treatment led to improvements in visual acuity among diabetic patients with OSA [17].

Choroidal perfusion may also play a critical role, particularly in AMD. Uslu et al. found that CPAP therapy increased choroidal thickness, a proxy for improved posterior segment circulation [4]. Since AMD involves hypoxia and inflammation at the level of the choriocapillaris, this suggests a plausible mechanism by which CPAP may reduce AMD risk. Furthermore, two systematic reviews by Kongchan et al. and Singh et al. demonstrated that CPAP does not adversely affect intraocular pressure and may support global ocular homeostasis, potentially minimizing glaucomatous and vascular insults in OSA patients [18,19].

By contrast, RVO risk is tightly coupled to thrombosis, systemic vascular disease, and local venous outflow dynamics; small case–control studies report higher OSA prevalence among RVO (especially CRVO) patients, but whether CPAP alters that trajectory remains uncertain and our EHR-level analysis was underpowered for rare events [6,20]. De Terán et al. showed that optic nerve structural damage in OSA was not reversed by CPAP, possibly explaining the limited impact on thrombotic retinal outcomes [21].

Similarly, database work suggests a modestly increased CSCR incidence in OSA, possibly mediated by sympathetic tone and corticosteroid pathways rather than hypoxic vascular injury; CPAP’s effect on CSCR has not been demonstrated, consistent with our null findings [7]. Fukutome et al. confirmed that CPAP use had no effect on CSC development during controlled sleep studies [22].

From a clinical standpoint, our findings underscore the potential of CPAP as a non-pharmacologic strategy to mitigate the risk of retinal vascular diseases in patients with OSA. Although the absolute reductions in incidence were modest, the widespread prevalence of OSA and associated retinal diseases implies meaningful public health relevance. Collaborative management between sleep medicine and ophthalmology may enhance early intervention strategies for retinal protection.

From a clinical standpoint, our findings underscore the potential of CPAP as a non-pharmacologic strategy to mitigate the risk of retinal vascular diseases in patients with OSA. Although the absolute reductions in incidence were modest, the widespread prevalence of both OSA and retinal conditions implies a substantial cumulative benefit at the population level. Importantly, CPAP therapy is already a mainstay treatment for OSA due to its effects on cardiovascular, metabolic, and neurocognitive outcomes. Given this, even modest ophthalmologic benefits may further justify its use. Collaborative management between sleep medicine and ophthalmology may enhance early intervention strategies. However, clinicians must also consider factors such as patient adherence, comfort, cost, and the multifaceted nature of retinal pathogenesis. In this context, retinal protection should be viewed as an added benefit rather than a primary indication for initiating CPAP.

While our findings align with pathophysiological insights and previous imaging-based and RCT data, it is essential to acknowledge that most of the supporting evidence remains indirect. Experimental and mechanistic studies specifically validating CPAP’s protective effects on the retina, particularly in human models, are still scarce and warrant further investigation.

### 4.2. Which Patients to Flag/Clinical Workflow

Clinicians managing DR or AMD risk should proactively ask about snoring, witnessed apneas, daytime sleepiness, and CPAP use, particularly in older, overweight, diabetic, or hypertensive patients in whom OSA prevalence is high but treatment adherence is variable. Documenting CPAP status in retina clinic intake could identify undertreated OSA that might contribute to systemic vascular dysregulation affecting the eye. Evidence linking OSA to DR progression and AMD development in large EHR cohorts supports integrating sleep history into ophthalmic risk stratification [2,3,4].

### 4.3. Limitations

This study has important limitations inherent to retrospective EHR analyses. First, causality cannot be inferred. Second, CPAP exposure was code-based; no device-verified adherence, nightly usage duration, pressure data, or longitudinal compliance measures were available, likely biasing associations toward the null. Third, retinal outcomes were identified by single ICD-10-CM codes without imaging confirmation, severity staging, laterality, or procedure linkage; miscoding and care received outside contributing systems could misclassify disease status. Fourth, the follow-up duration differed between exposure cohorts, raising the potential for ascertainment bias despite propensity matching. Fifth, key confounders, OSA severity metrics (apnea–hypopnea index), glycemic control, smoking, corticosteroid exposure, and ophthalmic treatment history were unavailable in the aggregate dataset. Finally, TriNetX participation is weighted toward large healthcare organizations; findings may not generalize to smaller or underserved settings. These constraints, common across published TriNetX ophthalmology studies, highlight the need for prospective, image-validated research with confirmed CPAP adherence.

## 5. Conclusions

In conclusion, this real-world cohort study shows that CPAP therapy is associated with a lower incidence of DR and AMD among adults with OSA. These protective effects may be mediated through improved oxygenation, enhanced microvascular integrity, and restored endothelial function. Further prospective, image-validated studies are warranted to confirm these findings and to guide integrated clinical approaches for retinal health in patients with sleep apnea.

## Figures and Tables

**Table 1:** Baseline characteristics after propensity score matching.

Variable	CPAP+ ^4^ (*n* = 50,877)	CPAP− (*n* = 50,877)	*p*-Value
Follow-up, mean ± SD ^1^ (days)	966.9 ± 1031.4	1106.5 ± 1111.0	
Follow-up, median (IQR ^2^) (days)	597 (1373)	744 (1434)	
Age at Index (years), mean ± SD	66.8 ± 13.8	66.9 ± 13.6	0.089
Male, n (%)	33,593 (66.0%)	33,606 (66.1%)	0.852
Hypertensive diseases, n (%)	41,936 (82.4%)	42,040 (82.6%)	0.443
Diabetes mellitus, n (%)	24,360 (47.9%)	24,240 (47.6%)	0.821
Chronic kidney disease, n (%)	18,185 (35.7%)	18,008 (35.4%)	0.211
BMI ^3^ (kg/m^2^), mean ± SD	29.3 ± 3.6	29.3 ± 3.2	0.284

^1^ SD: Standard Deviation; ^2^ IQR: Interquartile Range; ^3^ BMI: Body Mass Index; ^4^ CPAP: Continuous Positive Airway Pressure.

**Table 2:** Incidence of retinal outcomes.

Outcome	CPAP+ ^4^ (n, %)	CPAP− (n, %)	RD ^5^ (%)	RR ^6^ (95% CI ^7^)	OR ^8^ (95% CI)	*p*-Value
Diabetic Retinopathy	1611 (3.2%)	1748 (3.4%)	−0.3	0.922 (0.862–0.985)	0.919 (0.858–0.985)	0.016
CSC ^1^	33 (0.1%)	32 (0.1%)	0.0	1.031 (0.634–1.677)	1.031 (0.634–1.677)	0.901
AMD ^2^	1057 (2.1%)	1167 (2.3%)	−0.2	0.906 (0.834–0.983)	0.904 (0.831–0.983)	0.018
RVO ^3^	141 (0.3%)	158 (0.3%)	−0.0	0.892 (0.711–1.120)	0.892 (0.711–1.120)	0.325

^1^ CSC: Central Serous Chorioretinopathy; ^2^ AMD: Age-Related Macular Degeneration; ^3^ RVO: Retinal Vein Occlusion; ^4^ CPAP: Continuous Positive Airway Pressure; ^5^ Risk Difference; ^6^ Risk Ratio; ^7^ CI: Confidence Interval; ^8^ Odds Ratio.

## Data Availability

No new data were created.

## Abbreviations

The following abbreviations are used in this manuscript:

OSA	Obstructive Sleep Apnea
CPAP	Continuous Positive Airway Pressure
BMI	Body Mass Index
CSC	Central Serous Chorioretinopathy
AMD	Age-Related Macular Degeneration
RVO	Retinal Vein Occlusion
CKD	Chronic Kidney Disease
SD	Standard Deviation
IQR	Interquartile Range
CI	Confidence Interval
RR	Risk Ratio
OR	Odds Ratio
IRB	Institutional Review Board
HIPAA	Health Insurance Portability and Accountability Act
STROBE	Strengthening the Reporting of Observational Studies in Epidemiology
UMLS	Unified Medical Language System
ICD-10-CM	International Classification of Diseases, 10th Revision, Clinical Modification
SNOMED	Systematized Nomenclature of Medicine
CPT	Current Procedural Terminology
